# ﻿*Smithiayehii* (Leguminosae, Papilionoideae), a new species from Taiwan

**DOI:** 10.3897/phytokeys.210.90598

**Published:** 2022-09-29

**Authors:** Chiu-Mei Wang, Chih-Yi Chang, Yen-Hsueh Tseng

**Affiliations:** 1 Department of Biology, National Museum of Natural Science, 1 Guancian Rd., Taichung 40453, Taiwan National Museum of Natural Science Taichung Taiwan; 2 Department of Forestry, National Chung-Hsing University, No. 145, Hsing-Ta Rd., Taichung 40227, Taiwan National Chung-Hsing University Taichung Taiwan; 3 Taiwan Forestry Research Institute, No. 53, Nanhai Rd., Zhongzheng Dist., Taipei City, 10066, Taiwan Taiwan Forestry Research Institute Taipei Taiwan

**Keywords:** endangered (EN), macro-morphology, pollen morphology, scanning electron microscopy (SEM), *Smithiaciliata* Royle, *S.sensitiva* Aiton

## Abstract

A new species of *Smithia* Aiton, *S.yehii* C.M.Wang, Chih Y.Chang & Y.H.Tseng, **sp. nov.** from the wetlands of Taiwan is reported in this article. This species was mistakenly identified as *S.sensitiva* Aiton, but can be distinguished by its pale yellow corolla (vs. vivid yellow), often smaller flowers and shorter style. There is also a color gradient on the adaxial surface of the leaflets between young and mature leaves. Surface sculpture of pollen of *S.yehii* has significantly larger perforations, and muri are wider than those of *S.sensitiva.* An identification key to the *Smithia* taxa of Taiwan and *S.sensitiva* is presented.

## ﻿Introduction

The genus *Smithia* Aiton belongs to the tribe Aeschynomeneae (Benth.) Hutch., Papilionoideae DC., Leguminosae Juss. (LPWG 2017) and contains c. 20 species (Sa and Delgado-Salinas 2010). The genus is widely distributed in the tropics, chiefly in Asia and Madagascar (Huang and Huang 1987; Klitgaard and Lavin 2005). There are fourteen species in India (Balan and Predeep 2017), five species in China (Sa and Delgado-Salinas 2010), and two species in Taiwan (Huang and Ohashi 1977; Huang and Huang 1987, 1993).

The first record of Taiwanese *Smithia* was made by Forbes and Hemsley (1887), in which they recorded the species, *S.sensitiva* Aiton. Next, Hayata (1911) described a new species, *S.nagasawai* Hayata, based on its truncated or round apex of bracts, which differ from the acute apex of bracts in the similar *S.ciliata* Royle. Later, Hosokawa (1936) recorded *S.ciliata* in central Taiwan. Huang and Ohashi (1977) treated *S.nagasawai* as a synonym of *S.ciliata*. Since then, all authors have treated only two species of *Smithia* in Taiwan in subsequent papers (Huang and Ohashi 1977; Huang and Huang 1987, 1993): *S.sensitiva* and *S.ciliata*.

During a recent field and herbarium investigation, we noticed that the identity of *S.sensitiva* was somewhat controversial in Taiwan. Specimens initially identified in the field as *Smithiasensitiva* had vivid yellow flowers up to 1.5 cm long with styles up to 8 mm long (Aiton 1789; Efloraofindia 2007 onwards; Sa and Delgado-Salinas 2010; Balan and Predeep 2017). However, all specimens previously identified as *S.sensitiva* in Taiwan had smaller pale yellow flowers with shorter styles. Hence, it was suspected the Taiwanese population was likely an unknown taxon distinct from *S.sensitiva.* The aim of the present study was to elucidate the taxonomic status of this taxon by morphological and palynological approaches.

## ﻿Materials and methods

### ﻿Morphological comparison

We compared three *Smithia* taxa including Taiwanese taxa, viz. *S.ciliata* and the unknown taxon, together with its similar species, viz. *S.sensitiva*, which were collected from herbaria (see additional specimens examined). Morphological measurements were made on both fresh and dried materials. For the morphological description, the terminology followed the studies of Sa and Delgado-Salinas (2010) and Balan and Predeep (2017).

### ﻿Herbarium resources

Herbarium acronyms followed Index Herbariorum (Thiers 2022, continuously updated). Voucher specimens collected for the current study were deposited in TCF and TNM. Physical or digital specimens from the following herbaria were examined: HAST, PH, TAI, TAIE, TAIF, TCF and TNM. Type information of *S.sensitiva* followed the study of Balan and Predeep (2017).

### ﻿Pollen morphology

We compared the pollen morphology of the unknown taxon with that of its similar species, *S.sensitiva*, and information about the voucher specimens is provided in Table 1. Pollen materials were treated according to the methods of Schols et al. (2004) and Halbritter (1998). Pollen grains were obtained from herbarium materials and isolated anthers were rehydrated overnight. Whole anthers were fixed in 2% glutaraldehyde overnight, then treated with DMP (2, 2-dimethoxypropane) for 30 minutes, and transferred to acetone for 30 minutes before critical-point drying (CPD). Dried pollen was mounted on a stub and sputter coated with gold for > 100 s (Quorum SC7620) and examined by scanning electron microscopy (Hitachi S-3400N). The terminology for pollen shape, size, and exine ornamentation followed the recommendations of Erdtman (1952) and Halbritter et al. (2018).

**Table 1.** Specimens referenced for *Smithiayehii* C.M.Wang, Chih Y.Chang & Y.H.Tseng and *S.sensitiva* Aiton pollen morphology.

Taxon	Location	Coordinate	Altitude	Date	Voucher
*S.yehii*	Taiwan. Miaoli County Tunghsiao Township, Tunghsiao Township 14^th^ Cemetery	24.44718°N, 120.69563°E	81 m	17 Dec 2021	*Chih Y.Chang 3620* (TCF, TNM)
*S.sensitiva*	Thailand. Chiang Mai Province Samoeng district, Samoeng Forest	18.87321°N, 98.78213°E	1100 m	24 Nov 2018	*C.M.Wang 17941* (TNM)
	China. Guangdong Province Huidong County, Gutianshan Nature Reserve	23.19310°N, 114.78134°E	220 m	8 Sept 1984	*Huidong collector team 730* (TNM)

The quantitative palynological traits were measured and their means and standard deviations were calculated. For each quantitative character, the Shapiro-Wilks normality test was first used to check the distribution, then an independent sample *t*-test was performed after logarithmic transformation (Kim 2015). All analyses were done using the PASW Statistics ver. 18 software (Sarma and Vardhan 2018).

### ﻿Distribution map

The occurrence data was based on herbarium specimens. A distribution map was generated by using the package of Lin (2018) for QGIS ver. 3.4.

## ﻿Results and discussion

**Figure 1.**
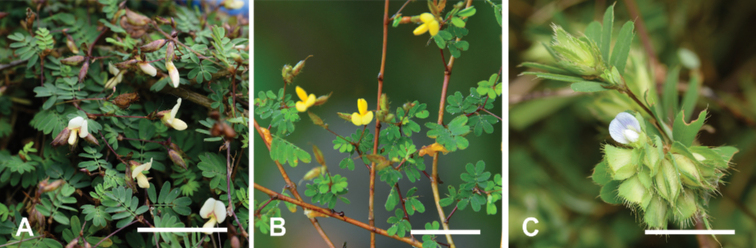
Comparison of *Smithiayehii* C.M.Wang, Chih Y.Chang & Y.H.Tseng and its similar species. Scale bars: 3 cm **A***S.yehii* (photo by C.M.Wang, from Miaoli, Taiwan) **B***S.sensitiva* Aiton (photo by Chih Y.Chang, from Chiang Mai, Thailand) **C***S.ciliata* Royle (photo by C.M.Wang, from Chiayi, Taiwan).

**Figure 2.**
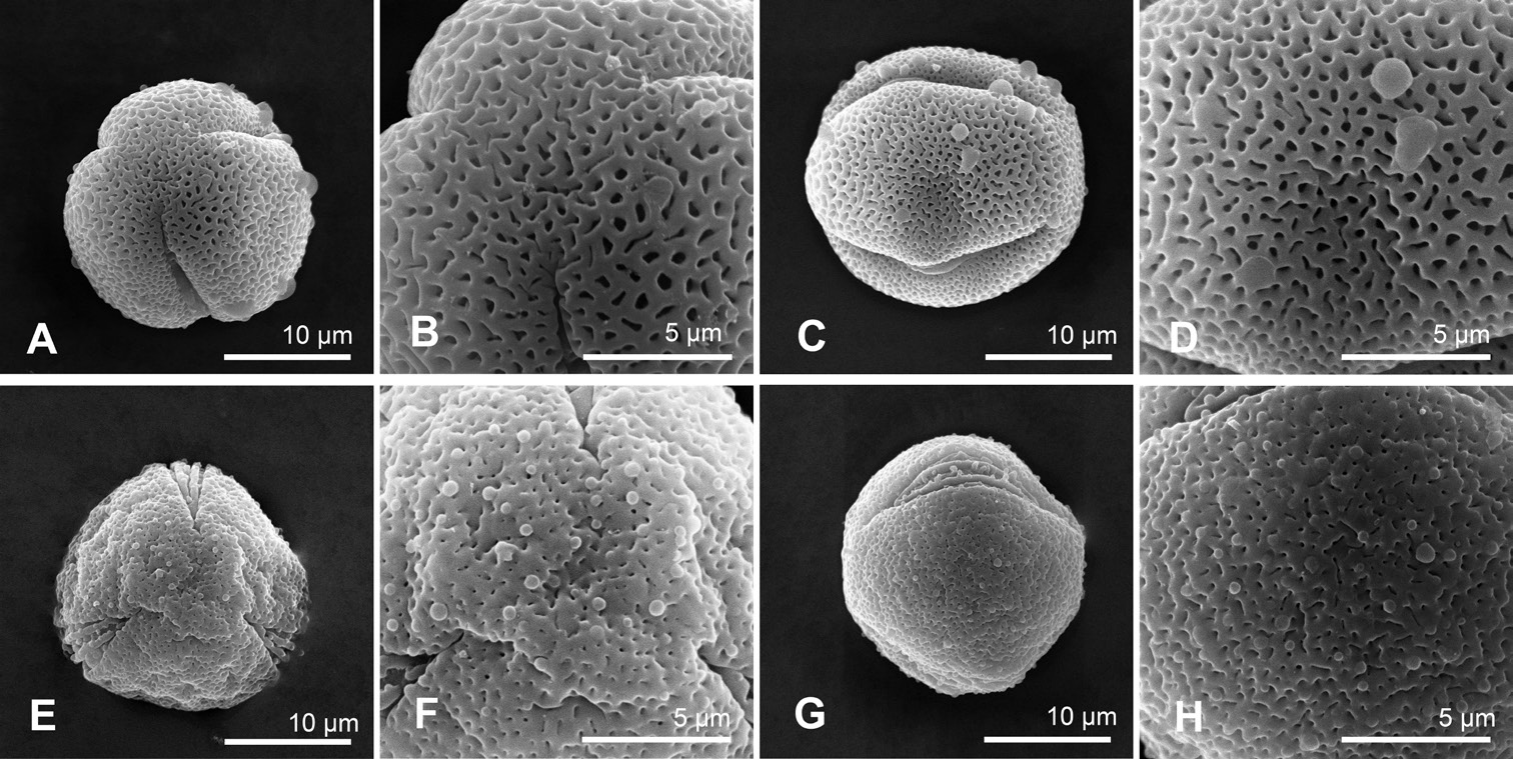
Comparison of the pollen morphology of *Smithia* Aiton **A–D***Smithiayehii* C.M.Wang, Chih Y.Chang & Y.H.Tseng **E–H***S.sensitiva* Aiton **A, E** polar view **B, F** exine ornamentation of polar view **C, G** equatorial view **D, H** exine ornamentation of equatorial view.

We compared the macro-morphology of the three *Smithia* taxa, *S.sensitiva*, *S.ciliata* and *S.yehii* (Fig. 1, Table 2) and the pollen morphology between *S.sensitiva* and *S.yehii* (Fig. 2, Table 3).

**Table 2.** Summary of diagnostic characters of *Smithiayehii* C.M.Wang, Chih Y.Chang & Y.H.Tseng and its similar species.

Characters	*S.yehii*	*S.sensitiva* (Efloraofindia 2007; Sa and Delgado-Salinas 2010; Balan and Predeep 2017)	*S.ciliata* (Huang and Huang 1993; Sa and Delgado-Salinas 2010)
Leaflet pairs	(2)4–9	4–11	4–7
Leaflet size	3.5–7.0 × 1.2–2.3 mm	4–15 × 2–3 mm	6–12 × 2–4 mm
Leaflets, adaxial color	dark green at apex, light green at base when young and mature	same color between apex and base	same color between apex and base
Flowers, number per raceme	1–7	3–6	12 to many
Flowers	0.7–1.0 cm long	0.8–1.5 cm long	c. 1 cm long
Calyx	entire at margin, scarious, with parallel veins	entire at margin, scarious, with parallel veins	ciliate at margin, membranous, with reticulate veins
Corolla	pale yellow	vivid yellow	white or yellow
Style	3.4–4.1 mm long	c. 8 mm long	c. 2.5 mm long
Pod shape and size	more or less straight, 0.5–0.8 cm long	more or less straight, c. 0.4 cm long	slightly orbicular, 1–1.5 cm long
Jointed number of pod	(4)6–7	4–6	6–8
Distribution	endemic to Taiwan, in wetlands and open places, at elevations of < 300 m	widely distributed in Australia, India, Madagascar and Tropical Asia, in field margins, wetlands; at elevations of < 1000 m	widely distributed in Taiwan, China, Bhutan, India, Japan, Malaysia, Nepal, Philippines, Thailand and Vietnam. Taiwan, in margin of thickets, at elevation of 1,000–1,800 m

**Table 3.** Comparison of pollen characters of *Smithiayehii* C.M.Wang, Chih Y.Chang & Y.H.Tseng and *S.sensitiva* Aiton.

Taxon	Polar axis length (μm)	Equatorial axis length (μm)	P/E ratio	Exine ornamentation	Murus wide (μm)	Perforate size (μm)
*S.yehii*	21.2±1.0 (19.6–22.7)	19.5±1.6 (16.5–21.9)	1.1±0.1 (0.9–1.3)	perforate	0.5±0.1 (0.3–1.1)	0.4±0.1 (0.2–0.6)
*S.sensitiv*a	21.0±1.4 (18.5–23.5)	18.4±1.6 (15.9–20.4)	1.1±0.1 (0.9–1.3)	perforate	0.4±0.1 (0.3–0.7)	0.2±0.1 (0.1–0.3)

### ﻿Macro-morphological differences

*Smithiaciliata* is distinctly different from other species in that its inflorescences often have more than twelve flowers (Fig. 1C), whereas those of *S.sensitiva* and *S.yehii* have fewer than seven flowers (Fig. 1 A, B). The calyx of *S.ciliata* is densely ciliate at the margin, and membranous with clearly reticulate veins, while *S.sensitiva* and *S.yehii* have entire margins and scarious parallel veins. The pods of *S.ciliata* are slightly orbicular and often more than 1.1 cm long, whereas both *S.sensitiva* and *S.yehii* are more or less straight and usually less than 1 cm long (Huang and Huang 1993; Sa and Delgado-Salinas 2010) (Table 2).

**Figure 3.**
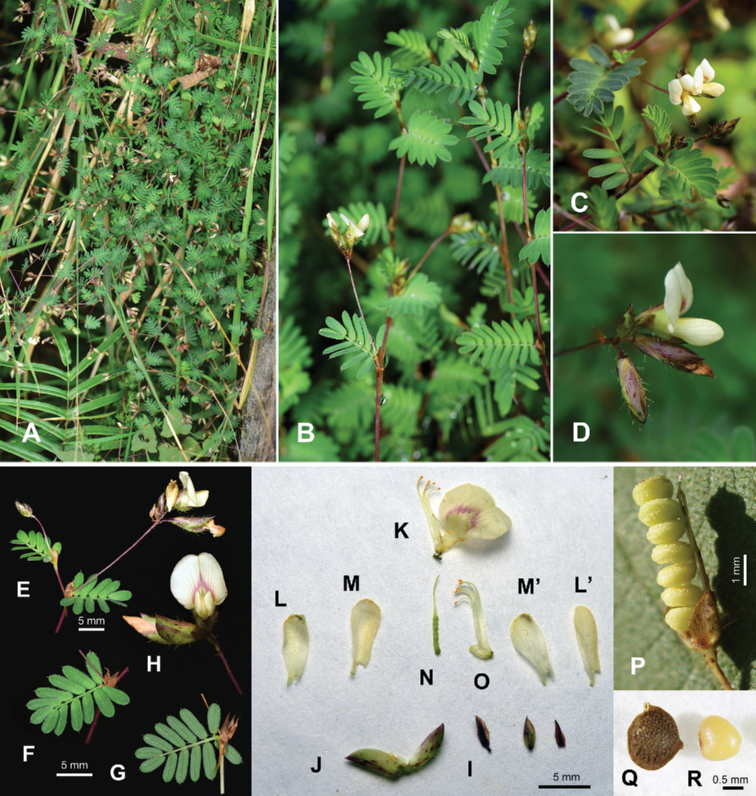
*Smithiayehii* C.M.Wang, Chih Y.Chang & Y.H.Tseng **A** habitat **B, C** habit **D, E** raceme **F** (leaf adaxial) **G** (leaf abaxial) **H** flowers **I** bracteoles **J** calyx **K** standard and part of diadelphous stamen **L** wing **M** keel **N** gynoecium **O** diadelphous stamen **P** pod **Q** joint of pod **R** seed. Voucher **A, D–H***Chih Y.Chang 3620* (TCF) **B, L–R***C.M.Wang 17247* (TNM).

Compared with *S.sensitiva*, the corolla of *S.yehii* is pale yellow (Fig. 1A); whereas *S.sensitiva* has a vivid yellow corolla (Fig. 1B). *S.yehii* often has smaller flowers (0.7–1.0 cm long) than *S.sensitiva* (0.8–1.5 cm long). In addition, *S.yehii* has a shorter style (3.4–4.1 mm) than *S.sensitiva* (c. 8 mm) (Efloraofindia 2007; Sa and Delgado-Salinas 2010) (Table 2). Leaves of *S.yehii* are usually smaller (3.5–7.0 mm long) with fewer than nine pairs of leaflets, while *S.sensitiva* often has up to eleven pairs of leaflets and they are larger (up to 1.5 cm) (Efloraofindia 2007; Sa and Delgado-Salinas 2010) (Table 2). Furthermore, *S.yehii* has color variations on parts of the adaxial surface of the leaflets, with dark green at the apex and light green at the base (Figs 1A, 3B, F); older leaflets are consistently dark green. *S.sensitiva* leaflets remain consistently pale green (Fig. 1B).

### ﻿Pollen morphological differences

The pollen grains of both *S.yehii* and *S.sensitiva* are small, tricolporate, and spheroidal with perforated exine ornamentation. *Smithiayehii* has significantly larger exine perforations (0.2–0.6 μm) than *S.sensitiva* (0.1–0.3 μm) (*p* = 0.000***), and *S.yehii* has significantly larger muri (width of 0.3–1.1 μm) than *S.sensitiva* (0.3–0.7 μm) (*p* = 0.044*) (Fig. 2, Tables 3, 4). The pollen characteristics also support the two taxa as distinct species.

**Table 4.** Students’ *t* scores and *p* values for quantitative characters of pollen grains.

Characters	t score	*p* value
Polar axis long	-1.753	0.095
Equatorial axis long	-1.687	0.107
P/E ratio	0.227	0.823
Interval between perforations	-2.076	0.044*
Perforation size	-7.361	0.000***

Note: *p* value significance: *p < 0.05, ** *p* < 0.01, ***p < 0.001

### ﻿Key to *Smithiayehii* and its similar species (modified from Huang and Huang (1993), and Sa and Delgado-Salinas (2010)

**Table:** 

1	Inflorescences often with more than 12 flowers, calyx ciliate at margin, membranous, with reticulate veins; pods slightly orbicular, more than 1.1 cm long	***S.ciliata***
–	Inflorescences with fewer than 7 flowers, calyx entire at margin, scarious, with parallel veins; pods more or less straight, less than 1 cm	**2**
2	Corolla pale yellow, flowers often less than 1 cm long (0.7–1.0 cm), style less than 5 mm long (3.4–4.1 mm); leaflets adaxial dark green at apex and light green at base between young and mature	***S.yehii***
–	Corolla vivid yellow, flowers up to 1.5 cm long (0.8–1.5 cm), style longer than 6 mm (c. 8 mm); adaxial surface of leaflets same color between apex and base	***S.sensitiva***

### ﻿Taxonomic treatment

#### ﻿ Smithia yehii

Taxon classificationPlantaeFabalesFabaceae

C.M.Wang, Chih Y.Chang & Y.H.Tseng sp. nov.

CC4F6444-03DF-594F-9188-5315CEA8721F

urn:lsid:ipni.org:names:77305894-1

**Figure 4.**
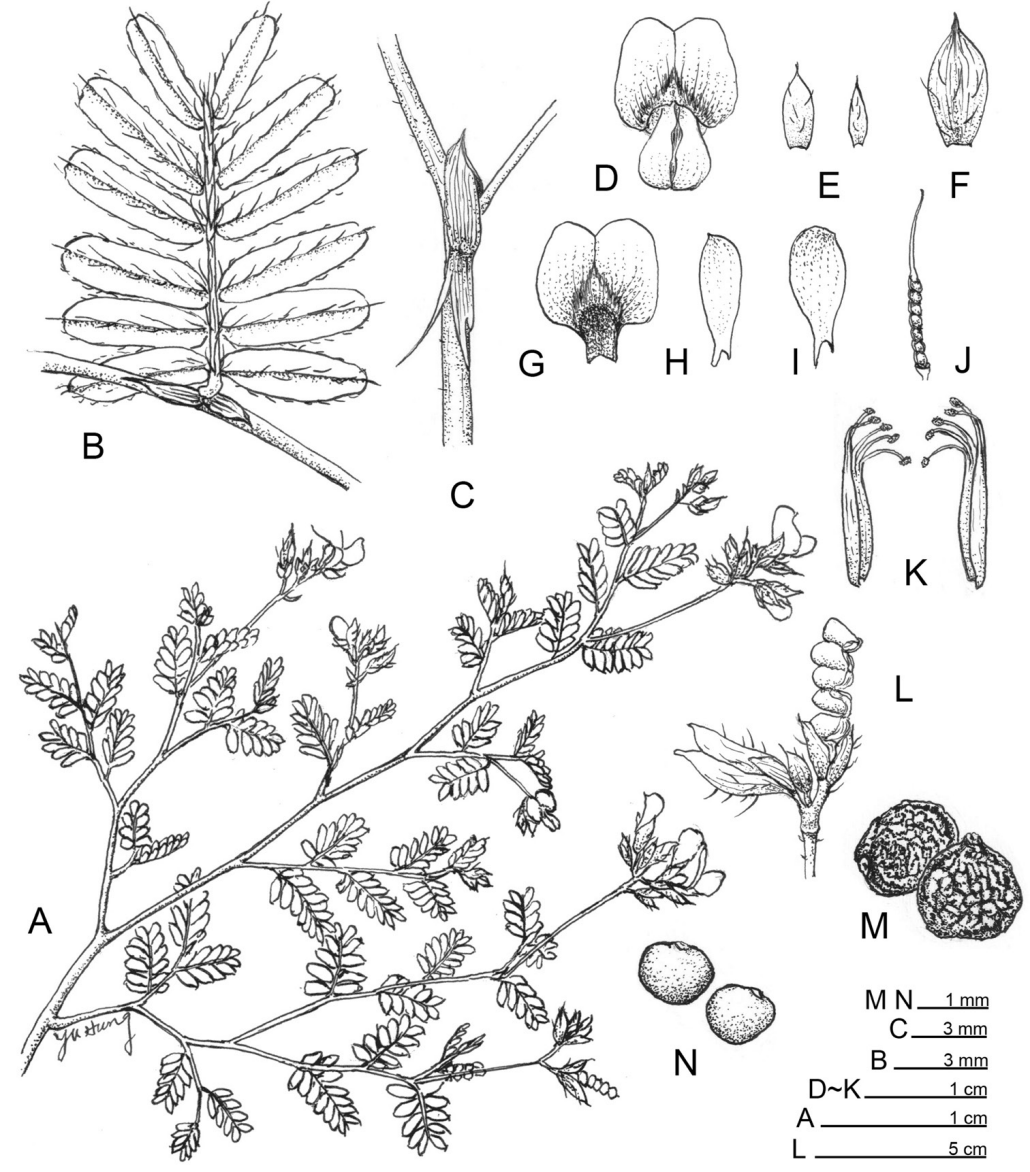
Line drawings of *Smithiayehii* C.M.Wang, Chih Y.Chang & Y.H.Tseng **A** habit **B** leaf (abaxial) **C** stipule **D** flower **E** bracteoles **F** calyx **G** standard **H** wing **I** keel **J** gynoecium **K** diadelphous stamen **L** pod **M** joint of pod **N** seeds.

**Figure 5.**
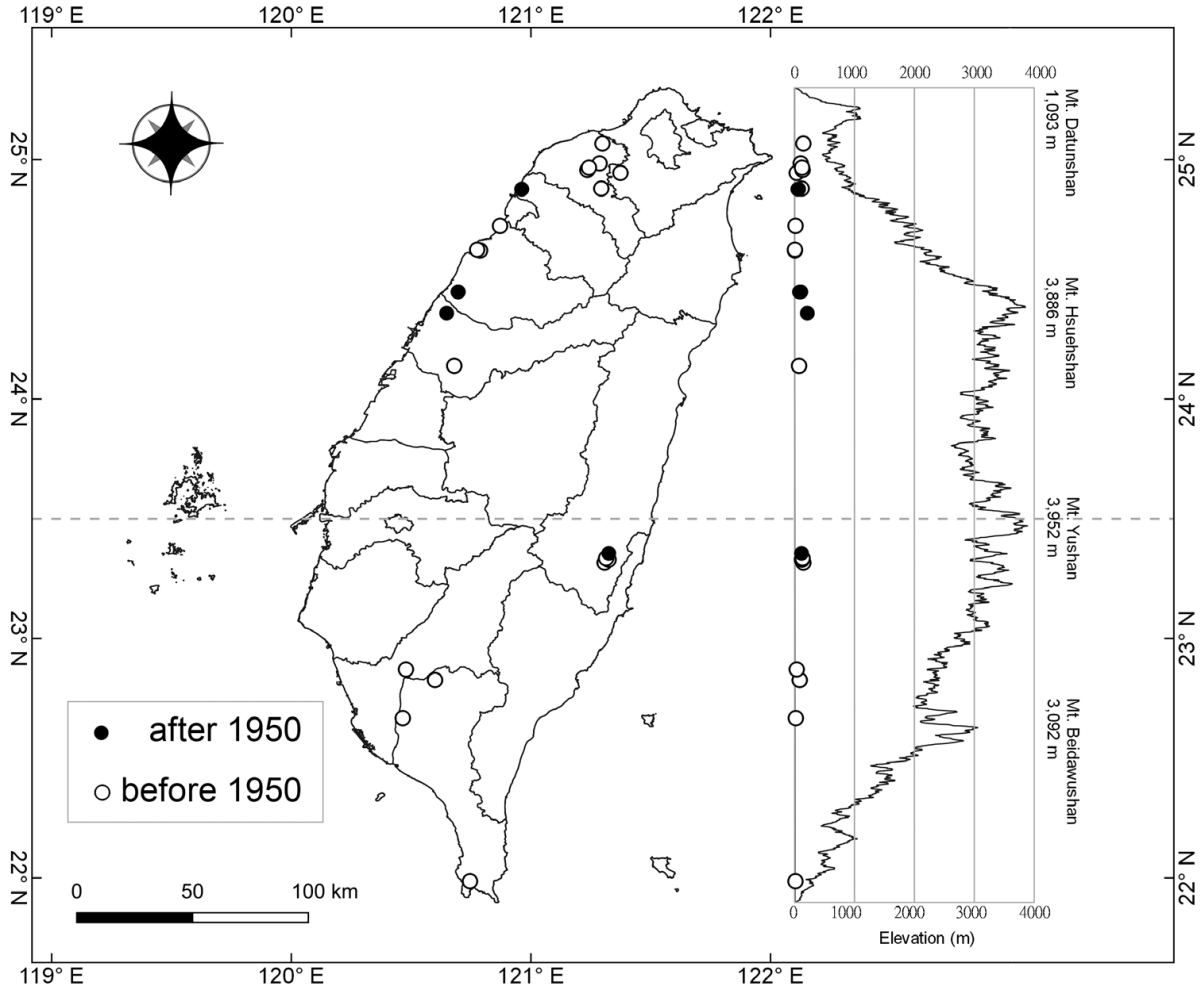
Distribution map of *Smithiayehii* C.M.Wang, Chih Y.Chang & Y.H.Tseng.

**Figure 6.**
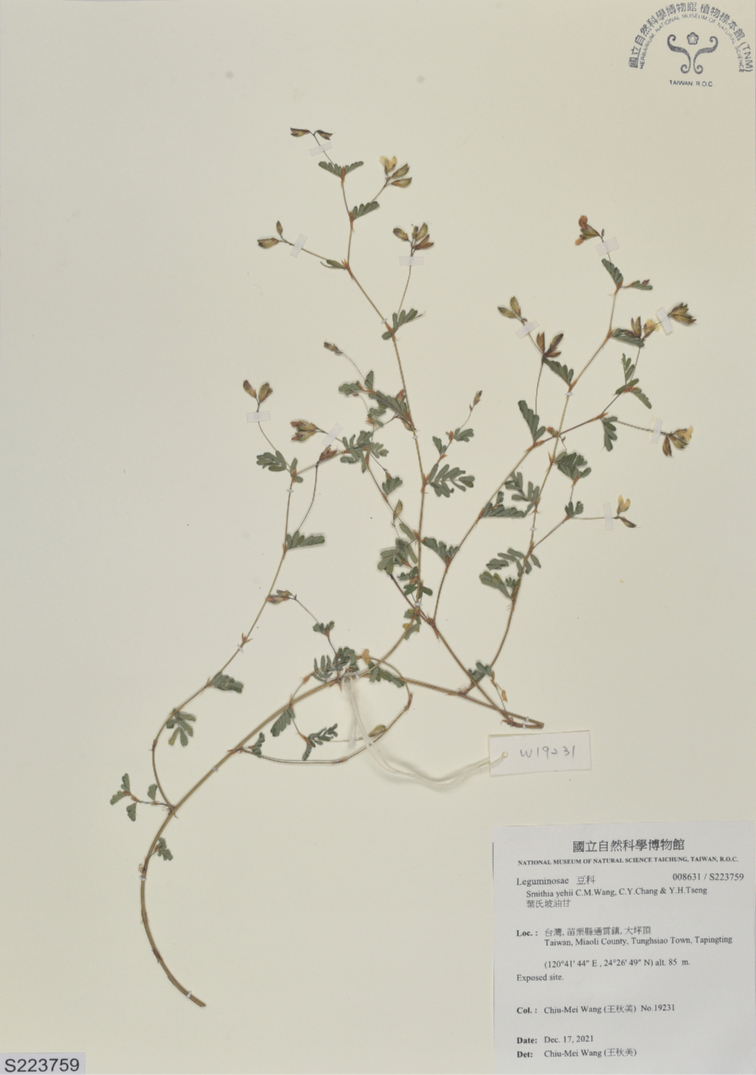
Holotype of *Smithiayehii* C.M.Wang, Chih Y.Chang & Y.H.Tseng.

Figs 1A , 2A–D , 3 , 4 , 5 , 6

S.sensitiva sensu acut. Forbes and Hemsley, J. Linn. Soc., Bot. 23: 170, 1887; Henry, List 32, 1896; Matsumura, Bot. Mag. (Tokyo) 16: 73, 1902; Hayata, Icon. Pl. Formosan. 1: 180, 1911; Hosokawa in Masamune, Short. Fl. Formosa 106, 1936; Chuang and Huang, Leg. Taiwan Past. 93, 1965; Huang and Ohashi in Li, Fl. Taiwan 3: 381, 1977; Huang and Huang, Taiwania 32(1): 88, 1987; Huang and Huang, Flora of Taiwan, 2^nd^ edition 3: 364, 1993, *non* Aiton.

##### Diagnosis.

The new species is similar to *S.sensitiva*, but can be distinguished by its pale yellow corolla (vs. vivid yellow), often smaller flower and shorter style, and color variation on adaxial surface of leaflets when young and mature, viz. dark green at apex and light green at base.

##### Type.

Taiwan. Miaoli County: Tunghsiao Township, Tunghsiao Township 14^th^ Cemetery, 81 m alt., 24.44718°N, 120.69563°E, 17 Dec 2021, *C.M.Wang 19231* (holotype: TNM) (Fig. 6).

##### Description.

Diffuse annual herb, 25–50 cm long; stem slender, sparsely bristly. Stipules 2.7–5.5 × 1.0–1.6 mm, ovate, striate, scarious, persistent; appendage to the stipules 1.9–3.6 mm long, bilobed. Leaf rachis bristly; petioles 0.9–1.6 mm long; leaflets (2)4–9 pairs, 3.5–7.0 × 1.2–2.3 mm, linear-oblong, obtuse at apex, mucronate, oblique and truncate at base, bristly beneath along the midvein and margins; adaxial surface dark green at apex, light green at base; older leaflets consistently dark green. Racemes axillary, 1.1–3.4 cm long, 1–7-flowered; peduncles filiform, sparsely bristly. Flowers 0.7–1.0 cm long; pedicels 1.0–3.1 mm long; bracteoles 2.3–4.0 × 0.9–2.4 mm, ovate, striate, persistent. Calyx parallel-veined, lips 4.5–8.2 mm long, equal, ovate, acute at apex, with a few scattered bristles. Corolla pale yellow, standard (5.2)6.2–9.0 × 5.7–8.0 mm, obovate, pale yellow with red circle pattern in centre; wings 4.0–6.9 × 1.8–2.6 mm, oblong, auricled; keels 4.8–7.5 × 1.9–2.5 mm, oblanceolate. Stamens diadelphous; filaments 5.1–6.4 mm long; anthers 0.2–0.3 mm long, ovoid. Ovary stipitate, 2.2–2.9 mm long, linear, (4)6–7-ovuled; style 3.4–4.1 mm long; stigma pointed. Pods more or less straight, 4.5–8.0 mm long, included, (4)6–7-jointed; joints 1.4–1.6 × 1.2–1.4 mm, papillose. Seeds 1.1–1.3 × 0.9–1.2 mm, reniform.

##### Phenology.

Flowering was observed from November to February and fruiting from December to March.

##### Distribution and habitat.

Endemic species of Taiwan. *Smithiayehii* grows in wetlands and open places, at elevations of < 300 m (Fig. 5). Common companion species are *Cirsiumlineare* (Thunb.) Sch. Bip. (Compositae), *Apludamutica* L. (Poaceae), *Eriochloavillosa* (Thunb.) Kunth (Poaceae), *Hydrocotylebatrachium* Hance (Araliaceae), and *Ampelopterisprolifera* (Retz.) Copel. (Thelypteridaceae).

##### Chinese name.

yè-shìh-po-yóu-gan (葉氏坡油甘).

##### Etymology.

The species epithet “*yehii*” was chosen to honor Prof. Mau-Shing Yeh (葉茂生), Department of Agronomy, National Chung-Hsing University, for his contributions to research into the legumes of Taiwan.

##### Palynology.

Pollen grains are small, tricolporate, and spheroidal, perforate in surface sculpture, and 19.6–22.7 × 16.5–21.9 μm, P/E ratio 0.9–1.3, perforations 0.2–0.6 μm in diam., and murus width 0.3–1.1 μm (Fig. 2A–D).

##### Conservation status.

*Smithiayehii* was evaluated as least concern (LC) by the Editorial Committee of the Red List of Taiwan Plants (2017) as *S.sensitiva*, because there were many records in the herbarium. However, many populations are probably extinct now. *Smithiayehii* is known after 1950 from only four sites (Fig. 5), each of which had only a few individuals (c. < 30) because of human disturbances and habitat fragmentation. Therefore, following the criteria of IUCN (2019), we regard this species as endangered (EN B2ab(ii, iii); C2a(i); D), and recommend that it urgently needs to be protected against extinction.

##### Specimens examined.

*Smithiayehii* C.M.Wang, Chih Y.Chang & Y.H.Tseng TAIWAN. **New Taipei City**: Sanxia District, “Ryoenpo” [Lungpuli], 24 Nov. 1910, 36 m alt., *T.Kawakami s. n.* (TAI!); **Taoyuan City**: “Toen” [Toyen], 25 May 1930, 134 m alt., *S.Suzuki 2627* (PH, TAI!); same loc., 17 Dec. 1933, 125 m alt., *S.Suzuki 4041* (TAI!); Daxi District, “Taikei” [Tahsi], 31 Mar. 1940, 119 m alt., *T.Nakamura 4311* (TAI!); Luzhu District, “Toen-Nankan” [Taoyuan-Nankan], 23 Nov. 1931, 147 m alt., *T.Suzuki 7876* (TAI!); **Hsinchu County**: Chupei City, Lienhua Temple, 19 June 1986, 63 m alt., *T.C.Huang 12692* (TAI!); same loc., 24 Oct. 1996, 57 m alt., *C.C.Huang 1619* (TAIE!); same loc., 30 Aug. 1996, 56 m alt., *K.C.Yang 4969* (HAST!); same loc., 16 Oct. 1997, 56 m alt., *Y.C.Kao 93* (HAST!); same loc., 29 Nov. 1997, 56 m alt., *W.C.Leong 667* (HAST!); same loc., 15 Sept. 1998, 68 m alt., *S.C.Liu 84* (TAIF!); same loc., 23 Aug. 1999, 56 m alt., *C.IPeng 17683* (HAST!); same loc., 30 Aug. 1996, 57 m alt., *K.C.Yang 4969* (TNM!); same loc., 15 Sept. 1998, 57 m alt., *S.C.Liu 84* (TNM!); **Miaoli County**: Houlong Township, “Koryu” [Houlung], 1 Nov. 1924, 6 m alt., *Y.Simada 1337* (HAST!, TAI!); Tunghsiao Township, Tunghsiao Township 14^th^ Cemetery, 27 Oct. 2016, 93 m alt., *T.C.Hsu 8660* (TAIF!); same loc., 21 Oct. 2017, 103 m alt., *L.H.Yang 908* (TAIE!); same loc., 2 Dec. 2017, 103 m alt., *R.P.Hsieh 49* (TAIE!); same loc., 26 Sept. 2019, 93 m alt., *T.C.Hsu 12070* (TAIF!); same loc., 20 Oct. 2021, 103 m alt., *Z.X.Chang 2666* (TAIF!); same loc., 11 Dec. 2017, 81 m alt., *C.M.Wang 17247* (TNM); same loc., 21 Oct. 2017, 81 m alt., *L.H.Yang 910* (TAIE!); same loc., 1 Mar. 2018, 81 m alt., *M.Y.Shen 5542* (TAIE!); same loc., 13 Oct. 2021, 81 m alt., *M.Y.Shen 6841* (TAIE!); same loc., 17 Dec 2021, *Chih Y.Chang 3620*, *3621*, *3622* (TCF); Zhunan Township, “Kityo” [Chiting], 3 Aug. 1940, 16 m alt., *Fukuya s. n.* (TAI!); **Taichung City**: “Taityushi” [Taichung], Oct. 1905, 75 m alt., *G.Nakahara s. n.* (PH); same loc., 27 Aug. 1931, 75 m alt., *S.Suzuki 8217* (TAI!); Dajia District, Mt. Tiehchen, 12 Oct. 1997, 213 m alt., *S.Y.Lu s. n.* (TAIF!); **Kaohsiung City**: Cishan District, “Banshoryo” [Chishan], 1 Nov. 1934, 36 m alt., *S.Suzuki 5825* (TAI!); **Pingtung County**: Gaoshu Township, “Takagi” [Kaoshu], 8 Nov. 1931, 86 m alt., *T.Hosokawa 3377* (TAI!); same loc., 8 Nov. 1931, 86 m alt., *T.Hosokawa s. n.* (TAI!); Hengchun Township, “Koshun” [Hengchun], Aug. 1915, 15 m alt., *E.Matuda 1083* (TAI!); Pingtung City, “Rokkwaiseki” [Liukueitsu], 31 Oct. 1934, 21 m alt., *S.Suzuki 5713* (TAI!); **Hualien County**: Yuli Township, “Tamazatosyo Nodyo” [Yuli], 28 Aug. 1933, 147 m alt., *Y.Yamamoto 3099* (TAI!); Hualien County, Yuli Township, “Tamazato” [Yuli], 29 Aug. 1933, 123 m alt., *Y.Yamamoto 3082* (TAI!); same loc., 29 Aug. 1933, 133 m alt., *Y.Yamamoto 3087* (TAI!); Yuli, 11 Feb. 1975, 116 m alt., *S.Y.Lu 3440* (TAIF!).

##### Additional specimens examined.

*Smithiasensitiva* Aiton Type: India. “India Orientalis” [Bengal], 1875, *Koenig s.n.*, (holotype: BM, photo!) China. **Fujian Province**: Wuping Country, Mt. Cuiye, 16 Oct. 2017, *T.W.Hsu 21882* (TAIE!); **Guangdong Province**: Huidong County, Gutianshan Nature Reserve, 220 m alt., 23.19310°N, 114.78134°E, 8 Sept. 1984, *Huidong collector team 730* (TNM!); Lianshan County, Shangshuai Town, Lungshuangshan, 150 m alt., 21 Oct. 1999, *F.Y.Zeng 2252* (TNM!); Lianshan County, Shangshuai Town, Lianguan Village, 500 m alt., 13 Oct. 2000, *H.G.Ye 5117* (TNM!); THAILAND. **Chiang Mai Province**: Samoeng district, Samoeng Forest, 1100 m alt., 18.87321°N, 98.78213°E, 24 Nov. 2018, *Chih Y.Chang 2139* (TNM); same loc., 24 Nov. 2018, *C.M.Wang 17941* (TNM); VIETNAM. **Lâm Đông Province**: Lac Duong District, Cong Troi Waterfall, Lat commune., 28 Oct. 2019, *T.C.Hsu 12222* (TAIF).

## Supplementary Material

XML Treatment for Smithia yehii
